# Chromosomal Fusions Facilitate Adaptation to Divergent Environments in Threespine Stickleback

**DOI:** 10.1093/molbev/msab358

**Published:** 2021-12-15

**Authors:** Zuyao Liu, Marius Roesti, David Marques, Melanie Hiltbrunner, Verena Saladin, Catherine L Peichel

**Affiliations:** 1 Division of Evolutionary Ecology, Institute of Ecology and Evolution, University of Bern, Bern, Switzerland; 2 Division of Aquatic Ecology and Evolution, Institute of Ecology and Evolution, University of Bern, Bern, Switzerland; 3 Department of Fish Ecology and Evolution, Centre for Ecology, Evolution, and Biogeochemistry, Swiss Federal Institute of Aquatic Science and Technology (EAWAG), Kastanienbaum, Switzerland; 4 Natural History Museum Basel, Basel, Switzerland

**Keywords:** adaptation, chromosomal fusion, natural selection, genome assembly, threespine stickleback, fourspine stickleback, Gasterosteidae

## Abstract

Chromosomal fusions are hypothesized to facilitate adaptation to divergent environments, both by bringing together previously unlinked adaptive alleles and by creating regions of low recombination that facilitate the linkage of adaptive alleles; but, there is little empirical evidence to support this hypothesis. Here, we address this knowledge gap by studying threespine stickleback (*Gasterosteus aculeatus*), in which ancestral marine fish have repeatedly adapted to freshwater across the northern hemisphere. By comparing the threespine and ninespine stickleback (*Pungitius pungitius*) genomes to a de novo assembly of the fourspine stickleback (*Apeltes quadracus*) and an outgroup species, we find two chromosomal fusion events involving the same chromosomes have occurred independently in the threespine and ninespine stickleback lineages. On the fused chromosomes in threespine stickleback, we find an enrichment of quantitative trait loci underlying traits that contribute to marine versus freshwater adaptation. By comparing whole-genome sequences of freshwater and marine threespine stickleback populations, we also find an enrichment of regions under divergent selection on these two fused chromosomes. There is elevated genetic diversity within regions under selection in the freshwater population, consistent with a simulation study showing that gene flow can increase diversity in genomic regions associated with local adaptation and our demographic models showing gene flow between the marine and freshwater populations. Integrating our results with previous studies, we propose that these fusions created regions of low recombination that enabled the formation of adaptative clusters, thereby facilitating freshwater adaptation in the face of recurrent gene flow between marine and freshwater threespine sticklebacks.

## Introduction

Understanding what facilitates rapid adaptation to new environments is of fundamental interest in evolutionary biology. A key question is whether adaptive loci are linked together in particular regions of the genome (Yeaman 2013; Schwander et al. 2014; Thompson and Jiggins 2014). Theoretical work has predicted that tight physical linkage between adaptive alleles would facilitate adaptation to divergent environments, particularly when there is gene flow, by preventing the production of unfit combinations of phenotypes through recombination (Charlesworth and Charlesworth 1979; Lenormand and Otto 2000; Hoffmann and Rieseberg 2008). In support of these theoretical predictions, empirical work from many systems shows that the distribution of adaptive loci across the genome is not random. For example, population genomic studies in many systems that show divergence despite the presence of gene flow have found that adaptive loci tend to be clustered in the genome, forming highly differentiated regions called “genomic islands” (Turner et al. 2005; Nadeau et al. 2012; Duranton et al. 2018; Irwin et al. 2018). Similarly, genetic linkage mapping studies have revealed evidence for the clustering of quantitative trait loci (QTL) underlying putatively adaptive phenotypes (Protas et al. 2008; Friedman et al. 2015; Peichel and Marques 2017).

Although these empirical findings support the theoretical predictions, it is still unclear how such QTL clusters and/or genomic islands form. Genomic clusters could evolve because of the higher probability of an adaptive mutation to fix near another locally adapted mutation since such architectures are seldom disrupted by recombination (the divergence hitchhiking hypothesis) (Feder et al. 2012; Via 2012). Genomic clusters could also be formed by genomic rearrangements that bring adaptive loci together (the genomic architecture change hypothesis) (Yeaman and Whitlock 2011). A study incorporating both analytical models and individual-based simulations suggested that genomic clusters are more likely to form through genomic rearrangements that bring together adaptive loci than through the establishment of an adaptive mutation near another locally adapted mutation (Yeaman 2013). Consistent with this finding, empirical studies have often found that such genomic clusters are often associated with chromosomal rearrangements, such as inversions (Kirkpatrick and Barton 2006; Schwander et al. 2014; Thompson and Jiggins 2014; Wellenreuther and Bernatchez 2018). However, there are not many studies focusing on other kinds of chromosomal rearrangements, such as chromosomal fusions.

Unlike chromosome inversions, which can only create clusters by reducing recombination between loci that are already physically linked, chromosomal fusions have been predicted to facilitate adaption both by bringing together previously unlinked loci and by changing the recombination landscape to create a new region of reduced recombination (Guerrero and Kirkpatrick 2014). Chromosomal fusions (and fissions) are common, as evidenced by the dramatic differences in chromosome number among species. Across multicellular eukaryotes, diploid chromosome number ranges from 2 to 1,260 (Sinha et al. 1979; Crosland and Crozier 1986). Chromosome numbers can even vary between closely related species (Wang and Lan 2000; Lysak et al. 2006; Ross et al. 2009; Urton et al. 2011; Valenzuela and Adams 2011) or be polymorphic within species (Dobigny et al. 2017; Wellband et al. 2019). Robertsonian fusions (i.e., fusions between two acrocentric chromosomes at their centromeres) are the most common type of chromosomal rearrangement in plants and animals (Robinson and King 1995). These Robertsonian fusions can have profound impacts on the recombination landscape across the entire genome (Vara et al. 2021). These effects are most obvious on the Robertsonian chromosomes, where recombination is restricted to the distal ends of the chromosome in fusion heterozygotes as well as in fusion homozygotes (Bidau et al. 2001; Castiglia and Capanna 2002; David and Janice 2002; Franchini et al. 2016, 2020; Vara et al. 2021). More generally, chromosomal fusions create larger chromosomes, which have a lower average recombination rate (Roesti et al. 2013; Haenel et al. 2018; Cicconardi et al. 2021). Despite this clear impact of chromosomal fusions on recombination, there is little empirical evidence supporting the hypothesis that chromosomal fusions play a role in adaptation (but see Kitano et al. 2009; Bidau et al. 2012; Wellband et al. 2019).

**Fig. 1.**
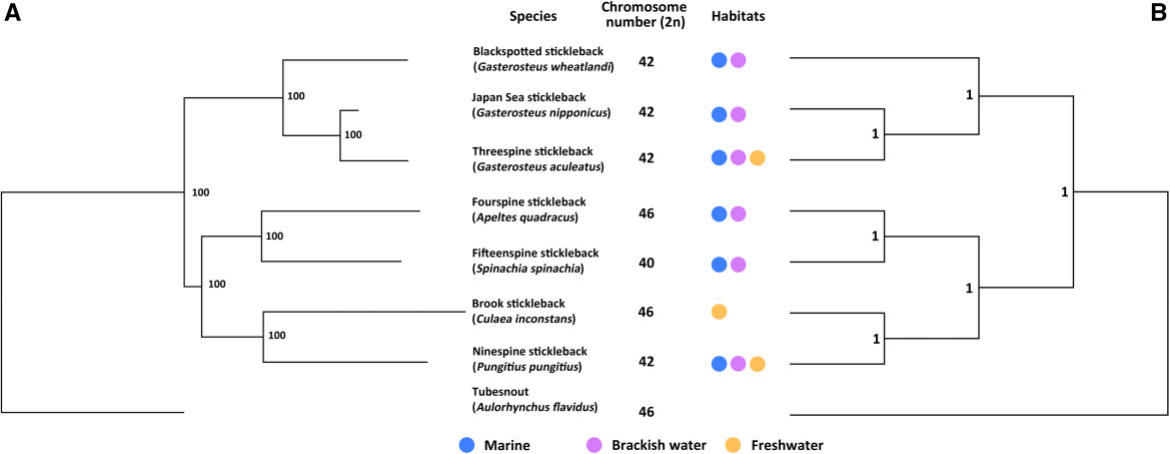
Phylogeny of stickleback species and the *Aulorhynchus flavidus* outgroup. (*A*) Phylogenetic relationship among species was reconstructed in RaxML using a concatenated supermatrix of 1,734 single-copy, orthologous genes. Numbers near nodes are bootstrap values. (*B*) Species tree was reconstructed in ASTRAL-III based on individual gene trees. Numbers near nodes are support values from ASTRAL-III. Data on diploid chromosome number are from Chen and Reisman (1970), Ocalewicz et al. (2008), Ross and Peichel (2008), Kitano et al. (2009), Ross et al. (2009), and Ocalewicz et al. (2011) and this study for *Spinachia spinachia*, and data on habitats are from Wootton (1976) and Guo et al. (2019).

In this study, we used stickleback fish species in the family Gasterosteidae to examine whether chromosomal fusions have contributed to the formation of adaptive genomic clusters. This system provides an excellent opportunity to address the role of chromosome fusion in adaptation as closely related stickleback species differ in chromosome number (fig. 1). In particular, we focused on the fourspine stickleback (*Apeltes quadracus*), which has 23 pairs of chromosomes (2*n* = 46) and is primarily found in marine and brackish habitats, and the threespine stickleback (*Gasterosteus aculeatus*), which has only 21 pairs of chromosomes (2*n* = 42) and can live in freshwater as well as marine and brackish habitats (Chen and Reisman 1970; Wootton 1976; Ross and Peichel 2008; Ross et al. 2009; fig. 1). Previous studies have shown that the difference in chromosome numbers between *A. quadracus* and *G. aculeatu*s involves the large metacentric chromosomes 4 and 7 in *G. aculeatus*, which each represent two pairs of acrocentric chromosomes in *A. quadracus* (Urton et al. 2011). However, without data from a closely related outgroup species, it was impossible to determine whether there had been chromosomal fissions in *A. quadracus* or chromosomal fusions in *G. aculeatus.* However, it was intriguing to note that both chromosomes 4 and 7 have frequently been associated with QTL and genomic islands of divergence between marine and freshwater *G. aculeatus* (Hohenlohe et al. 2010; Jones et al. 2012; Roesti et al. 2014; Peichel and Marques 2017; Nelson and Cresko 2018; Fang et al. 2020; Magalhaes et al. 2021; Roberts Kingman et al. 2021), suggesting the possibility that chromosomal fusions might have facilitated adaptation to divergent habitats in this species. However, previous population genomic studies had not directly tested whether these chromosomes were specifically enriched for genomic clusters of adaptive loci.

Here, we generated a high-quality de novo assembly for *A. quadracus*, and then integrated comparative genomics and population genomics to address the following questions: 1) Is the difference in chromosome number between threespine stickleback (*G. aculeatus*) and fourspine stickleback (*A. quadracus*) due to chromosomal fusion in *G. aculeatus* or chromosomal fission in *A. quadracus*? 2) Is there an enrichment of QTL contributing to adaptive divergence in traits on chromosomes 4 and 7 in *G. aculeatus*? 3) Is there an enrichment of molecular signatures of divergent adaptation on chromosomes 4 and 7 in *G. aculeatus*? 4) How did chromosomal fusions facilitate adaptation to divergent habitats in *G. aculeatus*?

## Results and Discussion

### Phylogenetic Relationship and Chromosome Numbers of Stickleback Species

We generated phylogenetic trees for seven species of the Gasterosteidae family plus the outgroup species (*Aulorhynchus flavidus*) using 1,734 single-copy, orthologous coding gene sequences obtained from whole-genome sequencing data (*G. aculeatus*, *Pungitius pungitius*, *A. quadracus*, *Aul. flavidus*) and RNA-seq data (*G. nipponicus*, *G. wheatlandi*, *Culaea inconstans*, *Spinachia spinachia*) (supplementary table S1, Supplementary Material online). The phylogeny generated by concatenated sequences is highly supported with all bootstrap values equal to 100 (fig. 1*A*). It is consistent with a previous phylogeny generated from 11 nuclear genes and mitochondrial genomes (Kawahara et al. 2009). To account for incomplete lineage sorting, we also built a species tree. First, gene trees were reconstructed for each ortholog. Then, these trees were combined to find a topology that agrees with the largest number of quartet trees. The species tree is the same as the concatenated tree with high support values (fig. 1*B*).

Based on this phylogeny, it is likely that the ancestor of the Gasterosteidae family inhabited marine and brackish water. The brook stickleback (*C. inconstans*) is the only species that lives primarily in freshwater, whereas the threespine stickleback (*G. aculeatus*) and the ninespine stickleback (*P. pungitius*) are able to inhabit both marine and freshwater habitats, with the opportunity for gene flow between the marine and freshwater populations. Interestingly, these two species also have a diploid chromosome number of 42 (2*n* = 42), which is reduced relative to the diploid chromosome number (2*n* = 46) in the fourspine stickleback (*A. quadracus*), the brook stickleback (*C. inconstans*), and the outgroup *Aul. flavidus* (Li Q, Lindtke D, Rodríguez-Ramírez C, Kakioka R, Takahashi H, Toyoda A, Kitano J, Ehrlich RL, Mell JC, Yeaman S, personal communication). We also found that the fifteenspine stickleback (*S. spinachia*) has a lower diploid chromosome number (2*n* = 40) by counting metaphase chromosomes from three independent males (41 metaphases counted, mode 2*n* = 40, range 2*n* = 38–42) and three independent females (nine metaphases counted, mode 2*n* = 40, range 2*n* = 38–41; supplementary fig. S1, Supplementary Material online). Given that most teleosts have a diploid chromosome number of 48 or 50 (Naruse et al. 2004; Amores et al. 2014), it is likely that lower chromosome number in species within the stickleback family results from chromosomal fusions. However, it is also possible that the fusions were ancestral and that the greater number of chromosomes in some species results from chromosomal fission. To distinguish between these possibilities, we used the newly available whole-genome assemblies of the outgroup *Aul. flavidus* (Li Q, Lindtke D, Rodríguez-Ramírez C, Kakioka R, Takahashi H, Toyoda A, Kitano J, Ehrlich RL, Mell JC, Yeaman S, personal communication), *P. pungitius* (Varadharajan et al. 2019), and *G. aculeatus* (Nath et al. 2021), as well as the high-quality assembly of *A. quadracus* generated in this study. We then focused on the whole-chromosome rearrangements that have occurred in *G. aculeatus* to determine whether these rearrangements are associated with genetic loci that underlie adaptation to divergent marine and freshwater habitats in this species.

### De Novo Assembly and Annotation of the *A. quadracus* Genome

To generate a high-quality assembly of the *A. quadracus* genome, we used high-coverage PacBio long-read sequencing to assemble the genome of a female fish derived from a laboratory cross between two populations from Nova Scotia, Canada. Raw read coverage was 91.58× (39.2 Gb in total). 10× Genomics linked reads and HiC reads from the same individual were used for scaffolding the assembly separately. The final assembly is 428.91 Mb, and it contains 890 scaffolds, including 21 chromosome-level scaffolds. The N50 length is 18.10 Mb, and the assembly quality assessed by BUSCO was relatively high with 96.9% completeness. *Apeltes quadracus* has a smaller genome than the other existing stickleback genome assemblies (∼449 Mb for *G. aculeatus*; Nath et al. [2021] and ∼521 Mb for *P. pungitius*; Varadharajan et al. [2019]). We constructed a repeat library for *A. quadracus* using de novo and homology-based approaches (see Materials and Methods). After masking the repetitive regions, the rest of the genome was annotated with the evidence from RNA-seq data, homologous protein databases, and ab initio annotation. We filtered out annotated genes with poor quality (typically annotation edit distance [AED] >0.5), leading to 21,955 genes in the final version of the annotation. The accession numbers for the *A. quadracus* assembly and annotation are available in supplementary table S1, Supplementary Material online.

### Independent Fusions of the Same Chromosomes in *G. aculeatus* and *P. pungitius*

**Fig. 2.**
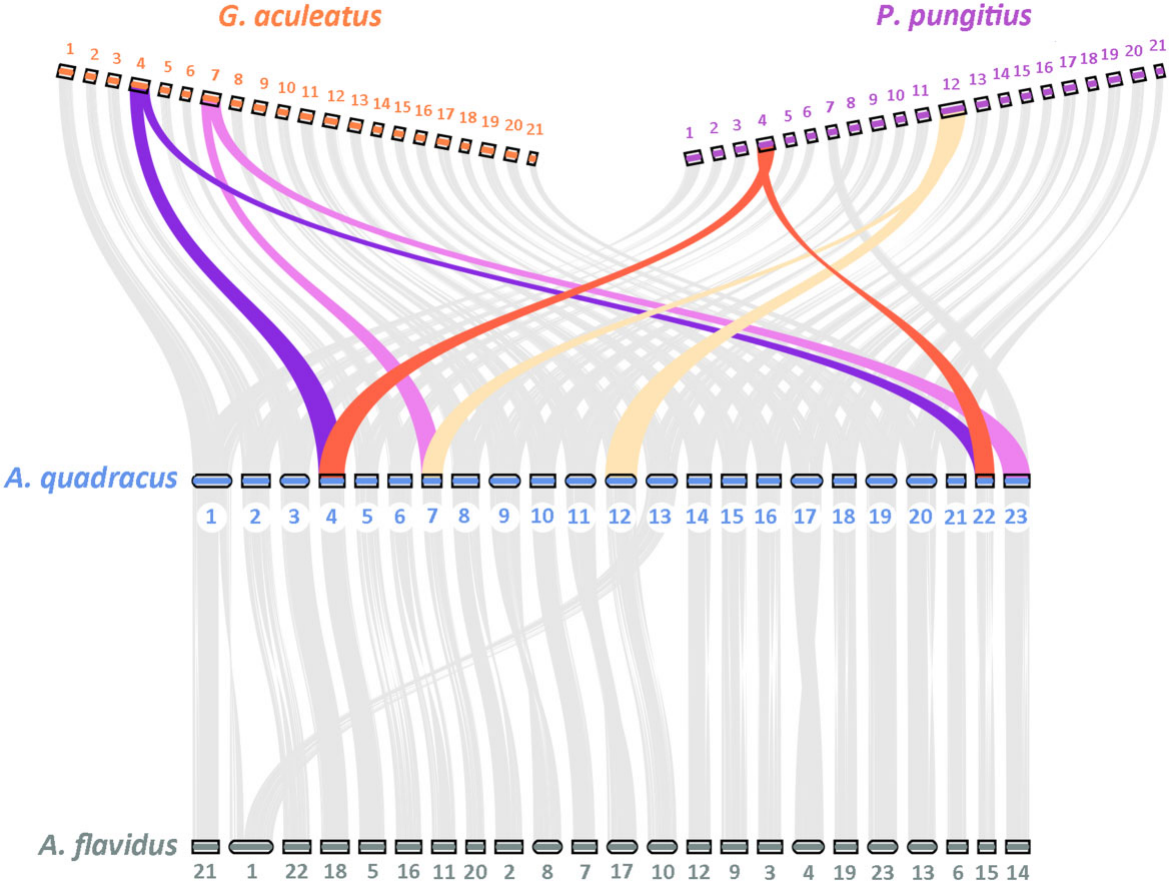
Synteny map of the *Aulorhynchus flavidus*, *Apeltes quadracus*, *Gasterosteus aculeatus*, and *Pungitius pungitius* genomes. The comparison is based on homologous coding region sequences. Colored rectangles are chromosomes and numbers indicate the corresponding chromosomes. Colored lines represent the fusion events in *G. aculeatus* and *P. pungitius*.

The difference in chromosome number between *G. aculeatus* (2*n* = 42) and *A. quadracus* (2*n* = 46) found in previous cytogenetic studies could either result from fission events in *A. quadracus* or fusion events in *G. aculeatus* (Ross et al. 2009; Urton et al. 2011). By comparing the genome assemblies of *G. aculeatus* and *A. quadracus*, as well as *P. pungitius*, to the outgroup species (*Aul. flavidus*), we conclude that two fusions occurred in *G. aculeatus* (fig. 2). The synteny map reveals that chromosomes 4 and 7 in *G. aculeatus* are likely the result of end-to-end fusions between chromosomes 4 and 22, and 7 and 23, respectively in *A. quadracus* (supplementary figs. S2–S4, Supplementary Material online). These four chromosomes are also unfused in the outgroup *Aul. flavidus*, which also has 23 chromosome pairs. Zooming into the detailed synteny map, we also find evidence for inversion and gene transposition between *A. quadracus* and *G. aculeatus* (supplementary figs. S2–S4, Supplementary Material online). On *G. aculeatus* chromosome 4, two large inversions have occurred near the fusion point. In contrast, the inversions on *G. aculeatus* chromosome 7 have occurred toward the chromosome ends. However, based on the order of the genes in the outgroup, these inversions have likely occurred in *A. quadracus*, not *G. aculeatus*.

Interestingly, chromosome 4 in *P. pungitius* is also the result of a fusion between *A. quadracus* chromosomes 4 and 22. However, taking the phylogeny (fig. 1) as well as a closer analysis of the fusion breakpoints into account (supplementary fig. S3, Supplementary Material online), the fusion events involving *A. quadracus* chromosomes 4 and 22 in both *G. aculeatus* and *P. pungitius* are likely to have occurred independently. Further, chromosome 12 in *P. pungitius*, which is the sex chromosome (Shapiro et al. 2009; Rastas et al. 2015; Natri et al. 2019) is the result of a fusion between *A. quadracus* chromosomes 7 and 12 (fig. 2). Although *A. quadracus* chromosome 7 is involved in fusion events in both *G. aculeatus* and *P. pungitius*, it has fused to different chromosomes in these species (fig. 2 and supplementary fig. S4, Supplementary Material online), again suggesting independent fusions have occurred in the two lineages. Together, these data demonstrate that chromosomal fusions have occurred in the two stickleback lineages that include species (*G. aculeatus* and *P. pungitius*) able to inhabit both marine and freshwater habitats, raising the possibility that such fusions have contributed to the ability of these species to adapt to divergent habitats in the face of gene flow.

### Enrichment of Marine-Freshwater QTL on Chromosomes 4 and 7 in *G. aculeatus*

**Fig. 3.**
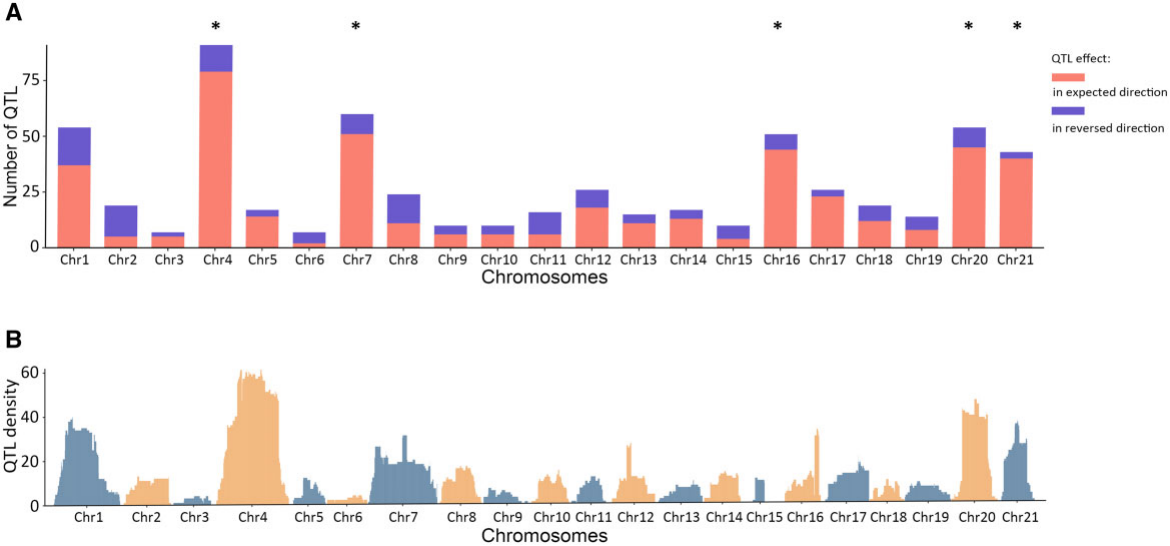
(*A*) Counts of QTL underlying traits that differ between marine and freshwater populations with QTL conferring an effect in the expected direction in red, and QTL conferring an effect in the reversed direction in purple. (*B*) Density of QTL confidence intervals mapped to the *Gasterosteus aculeatus* genome in 50-kb windows. QTL data are collected from previous studies (supplementary table S2, Supplementary Material online). Chromosomes with asterisks have significantly more QTL with effects in the expected direction than expected given either the number of genes on the chromosome or the chromosome length (supplementary table S2, Supplementary Material online).

If fusions facilitate adaptation by linking adaptive alleles, we would predict that an increased number of QTL underlying adaptive traits would map to the fused chromosomes, and that these QTL would have congruent effects in the expected direction (i.e., a marine allele confers a marine phenotype and vice versa) on multiple traits. Thus, we tested whether there was an enrichment of QTL with effects in the expected direction on *G. aculeatus* chromosomes 4 and 7 using a database of QTL identified in crosses between marine and freshwater populations (Peichel and Marques 2017). Indeed, we found that chromosomes 4 and 7, as well as chromosomes 16, 20, and 21, have significantly more QTL with effects in the expected direction than other chromosomes, accounting for variation in either the length of chromosomes or the number of genes on the chromosomes (fig. 3 and supplementary table S2, Supplementary Material online). Chromosome 21 has an inversion that is polymorphic within *G. aculeatus*, which is one of the strongest signals of divergence between worldwide marine and freshwater populations (Jones et al. 2012; Roesti et al. 2015; Fang et al. 2020; Magalhaes et al. 2021; Roberts Kingman et al. 2021). Although there are no apparent large-scale chromosomal rearrangements between marine and freshwater populations associated with chromosomes 16 or 20, the adaptive clusters on chromosomes 4, 7, and 21 are associated with chromosomal rearrangements that might facilitate linkage of adaptive traits.

### No Enrichment of Gene Transpositions or Gene Duplications on Chromosomes 4 and 7

It has also been proposed that such adaptive clusters could form via small-scale genomic rearrangements, such as transposition of single genes and/or gene duplications (Yeaman 2013). We therefore examined the distribution of gene duplication and gene transposition events in *G. aculeatus* relative to *P. pungitius*, *A. quadracus*, and *Aul. flavidus*. There were too few gene transposition events to determine whether the distribution of these genes varied among chromosomes. There are more gene duplications than expected on chromosomes 10, 11, 16, and 21, given either the length of the chromosome or the number of genes on the chromosome (supplementary table S3, Supplementary Material online). A comparison of the *G. aculeatus* and *Aul. flavidus* genomes also revealed no evidence for an enrichment of microrearrangements, lineage-specific genes, or gene duplications on *G. aculeatus* chromosomes 4 or 7, although gene duplications are enriched specifically within one region on chromosome 4 (Li Q, Lindtke D, Rodríguez-Ramírez C, Kakioka R, Takahashi H, Toyoda A, Kitano J, Ehrlich RL, Mell JC, Yeaman S, personal communication). It is therefore possible that gene duplication might also play a role in the formation of the QTL clusters on chromosomes 16 and 21, but not on the fusion chromosomes 4 and 7.

### Enrichment of Genomic Signatures of Selection on Chromosomes 4 and 7 in *G. aculeatus*

**Fig. 4.**
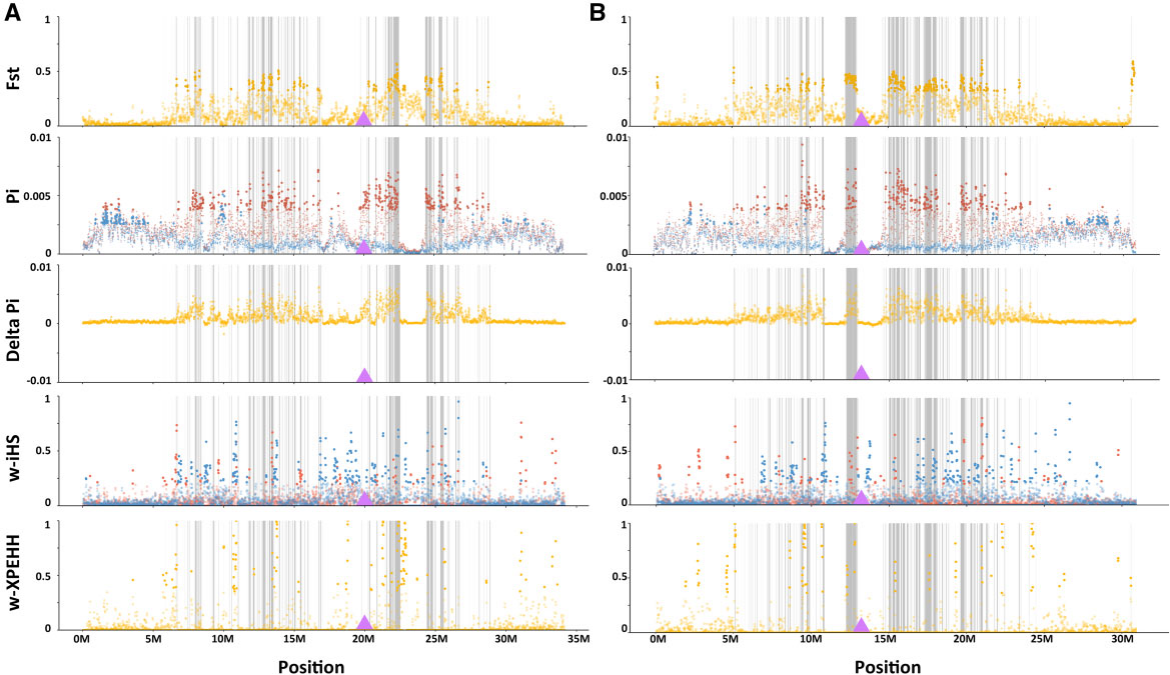
Signatures of selection in the Lake Washington freshwater and Puget Sound marine populations of *Gasterosteus aculeatus*. Statistics are shown here for chromosomes 4 (*A*) and 7 (*B*), with all chromosomes shown in supplementary figure S5, Supplementary Material online. All statistics were calculated in 20-kb sliding windows with a step size of 10 kb. Dark gray bars indicate the genomic islands and the purple triangle indicates the fusion points. From top to bottom: *F*_ST_ across the whole chromosome, with solid dots highlighting SNPs in the top 5% of genome-wide *F*_ST_; nucleotide diversity (Pi) of Lake Washington (red) and Puget Sound (blue) populations, with solid dots highlighting SNPs with the top 5% highest values of Pi in each population; differences of nucleotide diversity between the two populations. (Delta Pi=Pi_Lake Washington_−Pi_Puget Sound_); haplotype-based selection statistic iHS, with solid dots indicating the top 5% genome-wide outliers for Lake Washington (red) and Puget Sound (blue); and haplotype-based selection statistic XPEHH, with top 5% genome-wide outliers labeled in solid yellow dots.

The clustering of adaptive QTL on chromosomes 4 and 7 suggests that these chromosome fusions link adaptive loci together. However, from the QTL analysis, we can only observe this at the phenotypic level. To further explore whether chromosome fusions show signatures of selection at the sequence level, we examined different signatures of selection using whole-genome sequencing data. Using hidden Markov models (HMM), we identified genomic islands of differentiation between a marine (Puget Sound) and freshwater (Lake Washington) population of *G. aculeatus*. The distribution of genomic islands is uneven across the genome, and chromosomes 4, 7, 9, 11, and 20 have a significantly higher number of windows with outlier SNPs in genomic islands than expected, given either the length of the chromosome or the number of genes on the chromosome (for details of all enrichment analyses in this section, see Materials and Methods, supplementary fig. S5 and table S4, Supplementary Material online). Next, we used a window-based method to calculate *F*_ST_ across the genome. *F*_ST_ within genomic islands is elevated, and peaks are enriched on chromosomes 4 and 7 (fig. 4 and supplementary fig. S5, Supplementary Material online). For these two chromosomes, regions with elevated *F*_ST_ are found in the middle of the chromosomes. A similar pattern is also revealed by a topology weighting analysis (supplementary fig. S6, Supplementary Material online), in which regions in the middle of chromosomes 4 and 7 show a higher proportion of topology 1, indicating adaptation of freshwater populations.

We also calculated window-based nucleotide diversity (Pi) across the genome to trace the signature that selection left within each population. Overall, the nucleotide diversity of the Lake Washington freshwater population is higher than in the Puget Sound marine population, with delta Pi (Pi_Lake Washington_−Pi_Puget Sound_) always greater than 0. The greatest differences in nucleotide diversity between the populations are found on chromosomes 1, 4, 7, 20, and 21, with more diversity in the freshwater Lake Washington population (fig. 4 and supplementary fig. S5, Supplementary Material online). Within Lake Washington, there are more top 5% outlier windows for Pi than expected on chromosomes 4 and 7 (as well as on chromosomes 8, 20, and 21), particularly in the middle of the chromosomes (fig. 4 and supplementary fig. S5 and table S4, Supplementary Material online). Interestingly, genetic diversity in the regions under selection is lower in the Puget Sound marine population and elevated in the Lake Washington freshwater population (fig. 4 and supplementary fig. S5, Supplementary Material online).

**Fig. 5.**
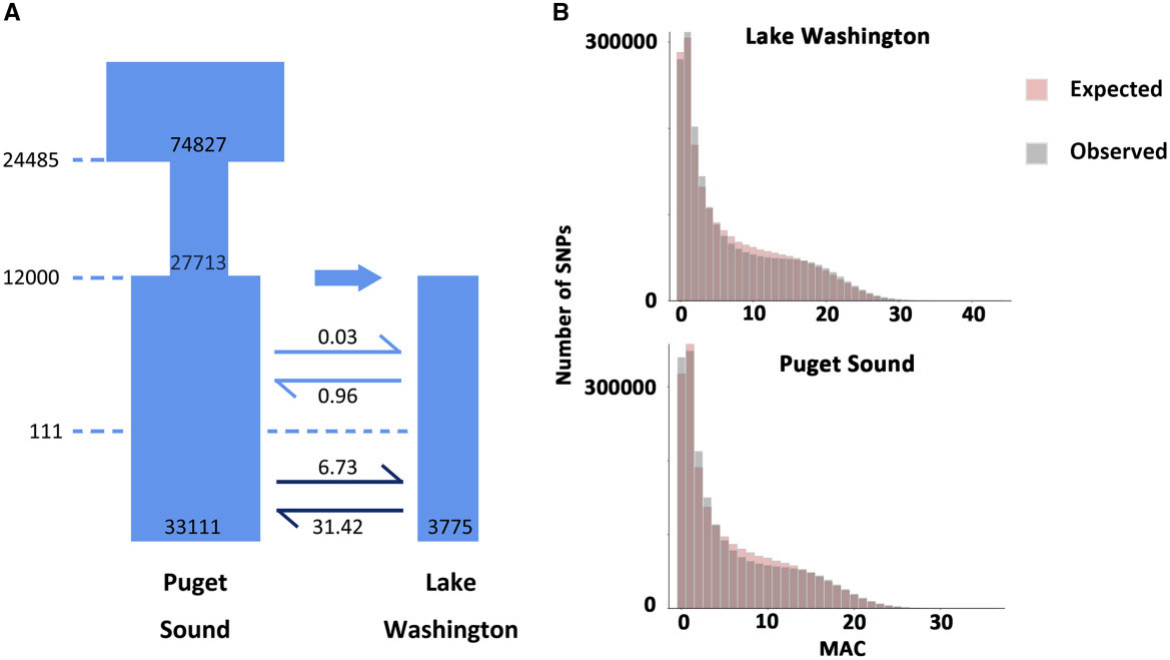
Demographic model of Lake Washington and Puget Sound populations. (*A*) Best demographic model inferred by fastsimcoal2. Dashed lines represent the time of the events. (*B*) Comparison of the observed minor allle count (MAC) spectrum (gray bars) and the simulated MAC spectrum (red bars).

The nucleotide diversity results are surprising. Most current-day freshwater populations of *G. aculeatus*, such as the Washington Lake population, were founded by marine stickleback after the end of the last ice age, approximately 12,000 years ago (Bell and Foster 1994). Thus, selection toward a novel environment is mainly thought to occur in the freshwater environment, leading to a reduction in genetic diversity near selected sites. Furthermore, freshwater populations are expected to have a smaller population size, where genetic drift would have a more powerful influence, leading to a faster loss of genetic diversity in the freshwater population. However, a recent simulation study has pointed out that gene flow can not only homogenize the genome but also increase diversity near regions under selection (Jasper and Yeaman 2020). To determine whether gene flow can explain the distribution of nucleotide diversity in our data, we built several demographic models (supplementary fig. S7, Supplementary Material online) to explore the most plausible evolutionary history of the Puget Sound marine and Lake Washington freshwater populations. Based on ΔAIC values, the best model has a bottleneck event in the ancestral population, followed by two reciprocal migration regimes (fig. 5 and supplementary table S5, Supplementary Material online). The effective population size in Puget Sound is 33,111, which is larger than the effective population size of 3,775 in Lake Washington, consistent with the expectation that the marine population has a larger population size. The inferred bottleneck is consistent with a previous pairwise sequentially Markovian coalescent inference of the demographic histories of these two populations (Shanfelter et al. 2019). Two migration regimes are inferred with an increase in migration at 111 years ago, which is roughly consistent with when the Lake Washington Ship Canal, which connects Lake Washington and Puget Sound, was built in 1917 (Edmondson 1991). During both periods of migration, the actual number of migrants from Puget Sound to Lake Washington is lower than the reverse, suggesting that more fish migrate from the freshwater environment to the marine environment. Overall, our demographic model suggests that migration between marine and freshwater populations is common, especially after the build-up of the Lake Washington Ship Canal. This is consistent with a scenario of gene flow increasing diversity near regions under selection (Jasper and Yeaman 2020) and our result that regions with high genetic diversity are associated with regions under selection. Similar results have been observed in Alaskan populations of *G. aculeatus*, with low genetic diversity in marine populations and high genetic diversity in freshwater populations in regions of the genome under divergent selection (Nelson et al. 2019). Their simulations suggest that this pattern results from asymmetries in population structure between the habitats, especially near locally adapted sites, and that this effect on diversity is strongest in regions of low recombination, such as we find on chromosomes 4 and 7.

Lastly, we used two haplotype-based methods to detect footprints of recent or ongoing selection. iHS is a statistic for detecting incomplete selective sweeps across the genome within a population (Voight et al. 2006), whereas XPEHH is a statistic for detecting (nearly) complete selective sweeps in one of two populations (Sabeti et al. 2007). We calculated the proportion of extreme values (w-iHS and w-XPEHH) in 20 kb windows with a step size of 10 kb. Signatures of recent selection exist across the whole genome in both populations, with more windows containing signatures of divergent selection (XPEHH) than expected between the populations on chromosomes 5, 9, and 17 (fig. 4 and supplementary fig. S5 and table S4, Supplementary Material online). Chromosomes 8 and 10 exhibit more windows of elevated iHS in Lake Washington, and chromosomes 4, 17, 18, and 21 exhibit more windows of elevated iHS in Puget Sound (supplementary fig. S5 and table S4, Supplementary Material online). Thus, these patterns of recent selection differ from the patterns nucleotide diversity and *F*_ST_, particularly on chromosomes 4 and 7 (fig. 4 and supplementary fig. S5, Supplementary Material online), consistent with previous results suggesting that most regions of strong divergence between marine and freshwater ecotypes are on the order of millions of years old (Nelson and Cresko 2018; Roberts Kingman et al. 2021).

### How Might Chromosomal Fusions Facilitate the Formation of Adaptive Clusters?

Overall, we find that signatures of divergent selection between marine and freshwater are distributed across the *G. aculeatus* genome, but that some regions of the genome show evidence for clustering of adaptive loci. The patterns we find in our population genomic analyses using whole-genome sequencing of a single marine-freshwater pair from the Eastern Pacific are consistent with the results of many population genomic studies, mostly using RAD-seq, in global marine-freshwater pairs (Hohenlohe et al. 2010; Jones et al. 2012; Roesti et al. 2014; Peichel and Marques 2017; Haenel et al. 2018; Nelson and Cresko 2018; Fang et al. 2020; Magalhaes et al. 2021; Roberts Kingman et al. 2021). In contrast to previous studies, we explicitly tested whether particular chromosomes are enriched for different signatures of selection. We found that chromosomes 4 and 7 have significantly more QTL associated with traits that diverge between marine and freshwater populations, more outlier SNPs in genomic islands of divergence, and higher levels of diversity in freshwater. By contrast these chromosomes do not have an excess of gene transposition or duplication events, or signatures of recent selection. These strong signals on chromosomes 4 and 7 have been previously observed, and they have been attributed to the fact that these are regions of low recombination (Roesti et al. 2014; Nelson et al. 2019; Roberts Kingman et al. 2021). Indeed, using genetic diversity as a proxy for recombination rate (Cicconardi et al. 2021), we find that chromosomes 4 and 7 have lower recombination rates than the unfused chromosomes in the *G. aculeatus* genome and that recombination rates on these chromosomes are lower than on their unfused homologs in *A. quadracus* (supplementary fig. S8, Supplementary Material online). Interestingly, there is an overall reduction in recombination on these two chromosomes relative to chromosome 1, which is also a large metacentric chromosome with similar patterns of reduced recombination across the middle of the chromosome (Roesti et al. 2013; Glazer et al. 2015; Shanfelter et al. 2019). This suggests that the reduction of recombination observed on chromosomes 4 and 7 is greater than we would predict for metacentric chromosomes of similar size. Furthermore, chromosome 1 does not show chromosome-wide enrichment for any signatures of selection or for QTL (supplementary fig. S5 and tables S2 and S4, Supplementary Material online). Thus, we hypothesize that the clustering of adaptive loci on chromosomes 4 and 7 is associated with the reduced recombination created by the chromosomal fusions.

There are two nonmutually exclusive hypotheses for how chromosomal fusions might facilitate adaptation (Guerrero and Kirkpatrick 2014). The first is that the fusion brings together pre-existing locally adapted alleles. The second is that the fusion creates a region of low recombination, which then enables the formation of adaptive clusters, as has been seen in the case of a chromosomal inversion in *Mimulus guttatus* (Coughlan and Willis 2019). In the case of the fusions found in *G. aculeatus*, it is difficult to determine whether one of these explanations may be most important, or whether both are playing a role. This is because the two sister species of *G. aculeatus* (*G. wheatlandi* and *G. nipponicus*) also have 21 pairs of chromosomes (fig. 1), and our preliminary assembly of a *G. wheatlandi* genome suggests that chromosomes 4 and 7 show the same arrangement as in *G. aculeatus*. Thus, the fusions were likely present in the common ancestor of the three *Gasterosteus* species. However, both *G. wheatlandi* and *G. nipponicus* can only live in marine or brackish habitats (fig. 1). Thus, the presence of the fusion itself was not enough to enable adaptation to freshwater. Previous work has suggested that duplications of the *Fads2* gene occurred in *G. aculeatus*, but not in *G. wheatlandi* or *G. nipponicus*, and that these duplications enabled *G. aculeatus* to take advantage of nutritionally depauperate freshwater habitats (Ishikawa et al. 2019). Interestingly, there is also a duplication of *Fads2* in *P. pungitius*, which can also live in freshwater. We speculate that once *G. aculeatus* (and perhaps *P. pungitius*) was able to invade freshwater, the region of low recombination created by the fusions provided a genomic region that could allow the buildup of adaptive alleles that were resistant to gene flow between marine and freshwater populations. Nonetheless, it is possible that the fusions we find in these species were fixed due to selection for linkage between alleles that provided an advantage in the ancestral habitat. A role for selection is suggested by convergent involvement of the same chromosomes in fusions in *Gasterosteus* and *Pungitius*. However, with our current data, we are unable to determine whether selection, drift, and/or another force like meiotic drive was responsible for the fixation of chromosomal fusions in sticklebacks (Dobigny et al. 2017).

Regardless of the mechanism of initial fixation, once fixed, we hypothesize that these fusions provided a unique genomic substrate for the formation of adaptive clusters in *G. aculeatus* as it was moving between marine and freshwater habitats during repeated bouts of glaciation and deglaciation during its evolutionary history over the past several million years. It does not appear that new genes were moving into these regions (Li Q, Lindtke D, Rodríguez-Ramírez C, Kakioka R, Takahashi H, Toyoda A, Kitano J, Ehrlich RL, Mell JC, Yeaman S, personal communication), and therefore they must have been built by what has been called “allele-only clustering,” which is when selection builds clusters of locally adapted alleles at loci already colocalized in the genome (Roesti 2018). The patterns of divergence we see indeed suggest that multiple adaptive clusters are embedded in the larger regions of particularly low recombination across chromosomes 4 and 7 (fig. 4 and supplementary fig. S8, Supplementary Material online). As many of these adaptive clusters in *G. aculeatus* (including those on chromosomes 4 and 7) are at least a million years old (Nelson and Cresko 2018; Roberts Kingman et al. 2021), there has been much time for the buildup of these adaptive alleles. Interestingly, older adaptive regions seem to be larger, suggesting that adaptive alleles are accumulating in these regions over time (Roberts Kingman et al. 2021). The accumulation of many adaptive alleles within these adaptive clusters is also consistent with a detailed study of the *Eda* region on chromosome 4, which showed evidence that multiple mutations within a 16-kb region of high divergence between marine and freshwater populations contribute to lateral plate and sensory lateral line phenotypes, and that linked mutations outside the *Eda* region are responsible for the QTL cluster observed on chromosome 4 (Archambeault et al. 2020). Taken together, these data are more consistent with the divergence hitchhiking hypothesis (Feder et al. 2012; Via 2012) than the genomic architecture change hypothesis (Yeaman 2013). Thus, our data suggest that even if the fusions themselves were not initially selected to link adaptive alleles, they have provided a genomic substrate that facilitates the process of divergence hitchhiking.

## Conclusion

Although the role of chromosomal rearrangements, such as inversions, in adaptation has been well-studied, the contribution of chromosomal fusions to adaptation is still unclear. By comparing genome assemblies, we found that two chromosomal fusions have occurred in *G. aculeatus*, and further demonstrate that these fused chromosomes are enriched in adaptive QTL and signatures of selection between marine and freshwater populations. We propose that these chromosomal fusions facilitated adaptation by altering the recombination landscape to create regions of low recombination that enabled the formation of adaptive clusters that can persist in the face of gene flow.

## Materials and Methods

### Ethics Statement

All experiments involving animals were approved by the Veterinary Service of the Department of Agriculture and Nature of the Canton of Bern (VTHa# BE4/16, BE17/17, and BE127/17).

### Sample Collections

In 2017, *A. quadracus* were collected from Rainbow Haven Beach (44.654857, −63.42113) and Canal Lake (44.498298, −63.90205) in Nova Scotia, Canada by Anne Dalziel. In 2018, *G. wheatlandi* were collected from Rainbow Haven Beach (44.654857, −63.42113) in Nova Scotia, Canada by Anne Dalziel. In 2017, *C. inconstans* were collected from the Sass River (60.073328, −113.312240) in the Northwest Territories, Canada by Julia Wucherpfennig; brains were dissected by Ian Heller and placed into RNAlater (Life Technologies, Carlsbad, CA). In 2018, *S. spinachia* were collected from the Baltic Sea (54.387423, 10.494736) near Hohenfelde, Germany by Arne Nolte.

### DNA and RNA Extraction and Sequencing

For assembly of the *A. quadracus* genome, DNA from a single laboratory-reared female resulting from a cross between a Rainbow Haven Beach female and a Canal Lake male (both from Nova Scotia, Canada) was used. High molecular weight DNA was extracted from the blood following (Peichel et al. 2020) and used to prepare a SMRTbell Express library for PacBio sequencing and a 10× Genomics library for Linked-Reads sequencing. The liver of the same individual was used to prepare a Hi-C sequencing library using the Phase Genomics Proximo Hi-C animal kit (Phase Genomics, Seattle, WA). Four SMRT cells were sequenced on a PacBio Sequel Platform, and the 10× Genomics and Hi-C libraries were sequenced for 300 cycles on an Illumina NovaSeq SP flow cell. To polish the PacBio reads, DNA from wild-caught individuals from Canal Lake (four females, four males) was extracted using phenol–chloroform and used to prepare Illumina DNA TruSeq libraries, which were sequenced for 300 cycles on an Illumina NovaSeq SP flow cell. All library preparation and sequencing were performed by the University of Bern Next Generation Sequencing Platform.

Total RNA was extracted from whole brains of wild-caught adult *G. wheatlandi* (four females, four males), *C. inconstans* (five females, five males), *A. quadracus* from Canal Lake (four females, four males), and *S. spinachia* (four females and four males) using Trizol (Life Technologies, Carlsbad, CA) following the manufacturer’s instructions. Illumina mRNA TruSeq libraries were prepared and either subject to 150-bp paired-end sequencing on an Illumina HiSeq3000 (*G. wheatlandi*, *C. inconstans*, *A. quadracus*) or 150-bp paired-end sequencing on an Illumina NovaSeq SP flow cell (*S. spinachia*) at the University of Bern Next Generation Sequencing Platform.

For this study, we also used the available genome assemblies for *G. aculeatus* (Nath et al. 2021), *P. pungitius* (Varadharajan et al. 2019), and the outgroup *Aul. flavidus* (Li Q, Lindtke D, Rodríguez-Ramírez C, Kakioka R, Takahashi H, Toyoda A, Kitano J, Ehrlich RL, Mell JC, Yeaman S, personal communication). We also used available RNA-seq data from *G. nipponicus* (Ishikawa et al. 2019). Supplementary table S1, Supplementary Material online, summarizes all samples and sequencing data used for this study and provides all relevant accession numbers.

### Reconstruction of the Stickleback Phylogeny

To determine if the phylogenetic relationships among the species in the Gasterosteidae family are consistent with previous studies using 11 nuclear genes and mitochondrial genomes (Kawahara et al. 2009), we built a phylogenetic tree using seven species in the family (*A. quadracus*, *C. inconstans*, *G. aculeatus*, *G. nipponicus*, *G. wheatlandi*, *P. pungitius*, *S. spinachia*) and an outgroup *Aul. flavidus*. For species with a reference genome (*A. quadracus*, *G. aculeatus*, *P. pungitius*, and *Aul. flavidus*), nucleotide and amino acid sequences of the coding regions were extracted. For species without a reference genome, we used RNA-seq data to build transcriptome assemblies.

RNA-seq reads were trimmed using Trimmomatic (v 0.36), and the reads were de novo assembled by the Trinity assembler (v 2.10.0). The open reading frames were predicted by Transdecoder (accessed on October 2, 2020) (Haas et al. 2013). Redundancy at the amino acid level was removed by cd-hit (v 4.8.1) (Li and Godzik 2006) with a threshold of 95% identity. Next, amino acid sequences of the eight species were compared with search for orthologs by OrthoFinder (v 2.3.12) (Emms and Kelly 2019), and only single-copy orthologs were kept for the downstream analysis. Then, we aligned amino acid sequences using muscle (v 3.8.1511) to guide the alignment of the corresponding nucleotides sequences. Sites with gaps or missing data were removed entirely, resulting in 1,734 alignments of single-copy orthologs. Phylogenies were built in two ways: 1) we concatenated alignments of 1,734 orthologs to build a supermatrix and reconstructed a phylogeny using RaxML (v8) (Stamatakis 2006); and 2) for each alignment, we first built gene trees in RaxML (v8) and then estimated the species tree using ASTRAL-III (V 5.7.4) (Zhang et al. 2018).

### Identification of Chromosome Number in *S. spinachia*

For the phylogenies shown in figure 1, we also added information on the known habitats of each species (Wootton 1976; Guo et al. 2019) and the diploid chromosome number (Chen and Reisman 1970; Ocalewicz et al. 2008, 2011; Ross and Peichel 2008; Kitano et al. 2009; Ross et al. 2009). However, there was no prior information on the diploid chromosome number for *S. spinachia*. We therefore used the protocol of Ross and Peichel (2008) to generate metaphase spreads from three of the *S. spinachia* females and three of the *S. spinachia* males used for the RNA-sequencing data (supplementary table S1, Supplementary Material online). Sex was determined by inspection of the gonads. The fish were euthanized in 0.2% tricaine methanesulfonate (MS-222), and the spleen was used for the metaphase spreads. Metaphase spreads from each individual were stained with DAPI and photographed on a Nikon Eclipse 80i microscope using a Photometrics CoolSNAP ES2 camera (Photometrics) and NIS-Elements BR 3.22.15 imaging software (Nikon, Japan). Chromosomes were counted from photos of individual metaphase spreads.

### *Apeltes quadracus* De Novo Genome Assembly

The PacBio assembly was generated using Flye 2.6 with default parameters (Kolmogorov et al. 2019), followed by the polishing step using Arrow (v 3.0) and Pilon (Walker et al. 2014) separately with default parameters in both cases. For polishing, whole-genome resequencing data described above from eight *A. quadracus* individuals (four males, four females) from Canal Lake, Nova Scotia, Canada (supplementary table S1, Supplementary Material online) were used. Raw reads were trimmed by Trimmomatic (v 0.36) (Bolger et al. 2014) with a sliding window of 4 bp. The first 13 bp of reads were dropped, and windows of the remaining reads were also dropped with an average quality score below 15. Genome size estimation was run by GenomeScope 2.0 (Ranallo-Benavidez et al. 2020) with trimmed data.

Contig scaffolding was conducted using the 10× Genomics linked reads and Hi-C proximity guided assembly separately. Contigs were linked by linked reads using ARCS (v 1.1.1) and LINKS (Warren et al. 2015; Yeo et al. 2018). Raw Hi-C reads were first processed with HiCUP (Wingett et al. 2015) and then assembled by Juicer (v. 1.5) (Durand et al. 2016) and 3D-DNA (v. 180922) (Dudchenko et al. 2017). After the first round of Hi-C scaffolding, the assembly was revised manually based on the contact map and then scaffolded again. The final step, gap-closing, was run by LR_Gapcloser (Xu et al. 2019). Assembly quality was evaluated by BUSCO v3 (Simão et al. 2015; Waterhouse et al. 2018).

### *Apeltes quadracus* Genome Annotation

The genome assembly was annotated in a two-step pipeline. The first step was the annotation of repeat elements. Miniature inverted-repeat transposable elements (MITE)-Tracker (Crescente et al. 2018) was used to detect MITE. Full-length long-terminal repeat (LTR) sequences were identified using LTR_finder (Xu and Wang 2007) and LTRharvest (Ellinghaus et al. 2008), and were further combined by LTR_retriever (Ou and Jiang 2018). Subsequently, RepeatMolder (v. 2.0) (Flynn et al. 2020) was used to identify novel repeat sequences. Libraries from MITE, LTR, and RepeatMolder were merged into a nonredundant library and passed to the final annotation of repetitive sequences with RepeatMasker (v. 4.0.9) (Smit et al. 2013).

The RNA-sequencing data generated from eight *A. quadracus* individuals (four males, four females) from Canal Lake, Nova Scotia, Canada (supplementary table S1, Supplementary Material online) and described above were used to aid in genome annotation. The raw reads were trimmed by Trimmomatic (v. 0.36) and then used as the input for Trinity assembler with default parameters (v. 2.10.0) (Grabherr et al. 2011).

The prediction and annotation of genes were conducted on the repeat-masked genome assembly with the Maker2 (v. 2.31.10) pipeline (Holt and Yandell 2011), including four rounds of annotation. In the first round, the transcriptome assembly generated by Trinity and protein data from *Danio rerio*, *G. aculeatus*, *P. pungitius*, *Takifugu flavidus*, and the Uniprot database (UniProt Consortium 2015) were used as evidence for the program. The second round of annotation included two training and prediction steps by AUGUSTUS (v. 3.2.3) (Stanke et al. 2008) and SNAP (Korf 2004). The results were then passed to MAKER2. For the third round annotation, GeneMARK-ES (Ter-Hovhannisyan et al. 2008) was combined with MAKER2. Finally, the second-round annotation was repeated with the resulting files from the third round. The final annotation was checked based on AED, and only annotations with AED score 0.5 or less were retained for downstream analysis. Functional annotation was conducted by eggnog-mapper (v2) (Huerta-Cepas et al. 2017).

### Genomic Synteny Analyses and Detection of Rearrangements between Species

Synteny analyses were conducted in two ways. First, Mummer4 and nucmer (Marçais et al. 2018) were used to compare the order of genes between *G. aculeatus* and *A. quadracus* on *G. aculeatus* chromosomes 4 and 7. Alignments shorter than 2,000 bp with an identity less than 85% were removed. Second, nonredundant coding sequence sets from four species (*G. aculeatus*, *A. quadracus*, *P. pungitius*, and *Aul. flavidus*) were used for cross synteny analysis. We used MCScan (Tang et al. 2008) in JCVI package to compare synteny on the chromosome level as well as the gene level. *Aul. flavidus* was chosen as the outgroup based on the phylogeny to examine whether the reduction of chromosome number in *G. aculeatus* and *P. pungitius* relative to *A. quadracus* is due to fission or fusion.

### Identification of Gene Transposition and Duplication Events

To detect gene duplication and transposition events, we first extracted single-copy orthologs from four species (*G. aculeatus*, *P. pungitius*, *A. quadracus*, *Aul. flavidus*) using OrthoFinder (v 2.3.12) (Emms and Kelly 2019). For gene duplication events, we used the duplication summary from OrthoFinder and focused on genes only duplicated in *G. aculeatus*; we included both intra- and interchromosomal duplications in the analyses. For gene transposition events, we focused on interchromosomal gene transpositions, in which a gene had moved to the focal chromosome in *G. aculeatus* from another chromosome in the other species. The homology of chromosomes from different species is based on our synteny map (fig. 2). If a gene is only present on a focal chromosome in *G. aculeatus* but is not present on the homologous chromosomes in other species, we considered it as a valid transposition event. The sex chromosome was excluded from these analyses.

To test whether any chromosomes had an excess of duplicated genes, the expected distribution of duplicated genes on each chromosome was calculated based on both the chromosome length in base pairs and the number of genes on the chromosome. The expected and observed distributions were compared in R through a goodness-of-fit test (chisq.test). Chromosomes with significantly higher values than expected were identified by standardized residuals with a value larger than 3 in both comparisons (supplementary table S3, Supplementary Material online). There were too few gene transposition events to analyze.

### Genomic Distribution of Marine-Freshwater QTL in *G. aculeatus*

To test if the fusion events in *G. aculeatus* are associated with clustering of adaptive traits, we used a modified version of a QTL database (Peichel and Marques 2017). The QTL data were filtered to remove redundant QTL following Rennison and Peichel (2022), and only the 655 QTL found in crosses between marine and freshwater populations were retained for the downstream analysis (supplementary table S2, Supplementary Material online). We first mapped all the retained QTL with confidence intervals to the *G. aculeatus* v.5 genome (Nath et al. 2021) in 50-kb windows, following Peichel and Marques (2017). Next, we used the data from the original QTL papers to determine whether the marine allele at these QTL confers a marine phenotype and vice versa, which would suggest that these QTL contribute to adaptation to the divergent marine and freshwater habitats. A chi-square test following (Peichel and Marques 2017) was used to test if the number of QTL with effects in the expected direction on a given chromosome is significantly different from the expected number of QTL with effects in the expected direction on that chromosome, given either the length of the chromosome or the number of genes on the chromosome. To identify significant deviations from the expectation on a particular chromosome, the standardized residuals for each chromosome were examined, with a value of 3 indicating the observed data is significantly larger than expected and a value of −3 indicated the observed data is significantly lower than expected (supplementary table S2, Supplementary Material online).

### Identifying Genomic Islands of Differentiation

Previous population genomic studies of marine-freshwater divergence were either based on very low coverage (2–5×) whole-genome sequence or RAD-seq data (Hohenlohe et al. 2010; Jones et al. 2012; Roesti et al. 2014; Nelson and Cresko 2018; Fang et al. 2020; Magalhaes et al. 2021; Roberts Kingman et al. 2021). To identify genomic islands of differentiation and signatures of selection between *G. aculeatus* marine and freshwater fish, we therefore used the only high-coverage (17–22×), whole-genome sequencing data available at the time of our analyses, which was from 25 freshwater individuals from Lake Washington and 24 marine individuals from Puget Sound (supplementary table S1, Supplementary Material online; Shanfelter et al. 2019). Trimmed reads (methods described as above) were mapped to the *G. aculeatus* v.5 genome assembly (Nath et al. 2021) by BWA (v 0.7.11) (Li 2013). Bam files were sorted with duplicates marked by Samtools (v 1.9) (Li et al. 2009) and MarkDuplicates in GATK4 (van der Auwera and O’Connor 2020) separately. Variants were called using HaplotypeCaller, and joint genotyping was conducted by combining all individuals for the population with GATK4 (van der Auwera and O’Connor 2020). For SNP filtration, we used Vcftools (0.1.16) and kept sites with minimum genotype qualities greater than 30, fewer than 20% missing genotypes, and a minor allele frequency greater than 0.05. To prevent bias caused by too high or too low sequencing depth, we also filtered out sites if the population mean depth coverages were less than half or greater than twice the average value for each population. Finally, sites that were not in Hardy–Weinburg equilibrium in each population were removed.

Using this data set, we followed the approach of (Hofer et al. 2012; Marques et al. 2016) to identify genomic islands of differentiation between the Puget Sound marine and Lake Washington freshwater populations of *G. aculeatus*. An HMM was used to find regions with exceptionally low and high divergence compared with the background divergence (assumed to be neutral). Only SNPs with minor allele frequencies >0.25 were used for this analysis because low-frequency allele SNPs tend to disrupt the detection of high differentiation regions which will never reach a high level of differentiation (Roesti et al. 2012). Locus level *F*_ST_ was estimated in Arlequin (v 3.5.2.2) (Excoffier and Lischer 2010), and outliers were identified assuming an infinite island model. An HMM method was run to model every chromosome separately based on the probability of an SNP being an outlier from the *F*_ST_ analysis. Scripts can be found at https://github.com/marqueda/HMM-detection-of-genomic-islands (last accessed July 17, 2020; Marques et al. 2016). Only regions passing the multiple-testing correction with a false discovery rate of 0.001 were recognized as “genomic islands.” We excluded chromosome 19, which is the *G. aculeatus* sex chromosome (Peichel et al. 2004) from the analysis.

### Detecting Signatures of Selection across the Genome

Scans for signatures of selection were performed between the Puget Sound marine and Lake Washington freshwater populations in various ways using the data set described above. A window-based *F*_ST_ distribution and nucleotide diversity were calculated with Vcftools (v 0.1.16) with a window size of 20 kb and a window step of 10 kb. To further identify selected regions, we also adopted haplotype-based statistics. We first extracted mapped reads with mapping quality larger than 20 and inferred haplotypes using WhatsHap (v1.0) (Martin et al. 2016) and shapeit4 (v 4.1.3) (Delaneau et al. 2019) with default parameters. Then, the output file was imported into the R package rehh (Gautier et al. 2017) to detect soft and incomplete sweeps within populations (iHS) and to detect complete sweeps that occurred in one population and not the other (XPEHH). We followed (Voight et al. 2006) to calculate the proportion of extreme iHS and XPEHH values (w-iHS and w-XPEHH, the proportion of |iHS| and |XPEHH| >2) in the same 20-kb overlapping windows. The sex chromosome, chromosome 19, was also excluded from this analysis.

To examine whether particular chromosomes were enriched for these signatures of selection, we compared the observed number of: 1) SNPs within genomic islands, 2) top 5% Pi outliers within each population, 3) top 5% |iHS| regions of outliers within each population, and 4) top 5% XPEHH regions of outliers on each chromosome to the expected numbers, given either the length of the chromosome or the number of genes on the chromosome in R through a goodness-of-fit test (chisq.test). Chromosomes with significantly higher values than expected were identified by standardized residuals with a value larger than 3 in both comparisons (supplementary table S4, Supplementary Material online).

### Topology Weighting Analyses

To explore the evolutionary histories of marine and freshwater alleles on the fusion chromosomes, we used a topology weighting approach. We built phylogenetic trees with the SNP data set for the genome scan in nonoverlapping windows for every 50 SNPs by RaxML (v8) (Stamatakis 2006) and conducted tree weighting in Twisst (Martin and Van Belleghem 2017). The analysis was performed on the two fused chromosomes, chromosomes 4 and 7, separately. For comparison, we performed the analysis on chromosome 1 because it is a large submetacentric chromosome with a similar length and recombination patterns as on chromosomes 4 and 7 (Urton et al. 2011; Roesti et al. 2013; Glazer et al. 2015; Shanfelter et al. 2019). However, it has not experienced interchromosomal fusion between the *G. aculeatus* and *A. quadracus* lineages.

### Inferring Demographic History

The SNP data set used for demographic simulations was the same as the one for detecting genomic islands with two differences. First, all rare alleles (i.e., a minor allele frequency <0.05) were kept. Second, we removed sites located in the genomic islands of differentiation. To account for linkage disequilibrium (LD), we used PLINK (v 1.9) to calculate and prune the SNP matrix to those with LD <0.1. To prevent bias from SNPs in repeated regions, we checked the distance between consecutive SNPs and discarded those where the distance was less than 5 bp.

To explore the evolutionary history of these two *G. aculeatus* populations and explain the patterns of genomic diversity, we reconstructed their demographic history with fastsimcoal2 (v 2.6) (Excoffier et al. 2013). The 1D folded observed site frequency spectrum (SFS) was calculated with easySFS (https://github.com/isaacovercast/easySFS, last accessed December 20, 2020) for each population. To maximize the number of segregating sites, 22 and 18 individuals of Lake Washington and Puget Sound were kept for downstream analyses respectively. We fixed the split time of freshwater and marine population to 12,000 years ago, assuming a generation time of 1 year (Bell and Foster 1994). Thirteen models were built to identify the best scenario (supplementary fig. S7, Supplementary Material online): 1) constant population size, 2) two bottlenecks while splitting, 3) two bottlenecks after splitting, 4) one bottleneck before splitting, 5) one bottleneck and splitting, 6) one bottleneck and splitting followed by a constant and reciprocal migration, 7) one bottleneck and splitting followed by an early reciprocal migration, 8) one bottleneck and splitting followed by a recent reciprocal migration, 9) one bottleneck and splitting followed by two reciprocal migration regimes, 10) one bottleneck and splitting followed by introgression from Lake Washington to Puget Sound, 11) one bottleneck and splitting followed by introgression from Puget Sound to Lake Washington, 12) one bottleneck and splitting followed by introgression from Lake Washington to Puget Sound and two reciprocal migration regimes, and 13) one bottleneck and splitting followed by introgression from Puget Sound to Lake Washington and two reciprocal migration regimes. To maximize the likelihood of each model, we randomly started from 100 parameter combinations in 50 expectation-conditional maximization cycles with a total of 200,000 coalescent simulations. A mutation rate of 7.9 × 10^−9^ was used, following Guo et al. (2013). For each model, we obtained the best likelihood values and estimated parameters from 100 optimizations. The best model was selected based on the smallest ΔAIC (supplementary table S5, Supplementary Material online).

### Genetic Diversity Analysis of Each Chromosome in Fused and Unfused Taxa

To explore whether fused chromosomes have a lower recombination rate, we compared genetic diversity of each chromosome in *G. aculeatus* and *A. quadracus*. Genetic diversity can be used as a proxy for recombination rate because a decrease in recombination rate should lead to an increase in levels of background selection and therefore decrease in genetic diversity. Such a relationship between genetic diversity and recombination rate has been observed in *Heliconius* butterflies (Cicconardi et al. 2021). To obtain diversity data in *A. quadracus*, the whole-genome resequencing data described above from eight individuals from Canal Lake, Nova Scotia, Canada (supplementary table S1, Supplementary Material online) were mapped by BWA (v 0.7.11) (Li 2013) to the *A. quadracus* reference genome generated in this study. Bam files were sorted with duplicates marked by Samtools (v 1.9) (Li et al. 2009) and MarkDuplicates in GATK4 (van der Auwera and O’Connor 2020) separately. Variants were called using HaplotypeCaller, and joint genotyping was conducted by combining all individuals with GATK4 (van der Auwera and O’Connor 2020). For SNP filtration, we used Vcftools (0.1.16) and kept sites with minimum genotype qualities greater than 30, fewer than 20% missing genotypes, and a minor allele count (MAC) greater than 2. For *G. aculeatus*, the same SNP data set for identifying genomic islands was used, except that we only used data from the marine population (Puget Sound) to prevent potential bias due to linkage to adaptive sites in the freshwater population. For both species, we extracted 4-fold degenerate sites with the script codingSiteTypes.py available at (https://github.com/simonhmartin/genomics_general, last accessed November 20, 2021). Genetic diversity was calculated in windows of 50 SNPs with the script popgenWindows.py (https://github.com/simonhmartin/genomics_general, last accessed November 20, 2021). The average value of each chromosome was calculated by hand, and genetic diversity on each chromosome was normalized relative to the average diversity of unfused chromosomes within a species.

## Supplementary Material

Supplementary data are available at *Molecular Biology and Evolution* online.

## Supplementary Material

msab358_Supplementary_Data
