# Supplementary material for: Chromosomal Fusions Facilitate Adaptation to Divergent Environments in Threespine Stickleback
Source: Mol Biol Evol. 2021 Dec 15;39(2):msab358. doi: 10.1093/molbev/msab358 (PMC8826639; doi:10.1093/molbev/msab358)
Supplement: msab358_Supplementary_Data [file msab358_supplementary_data.zip › Supplementary information_revised_submitted_MBE.pdf]

Supplementary information

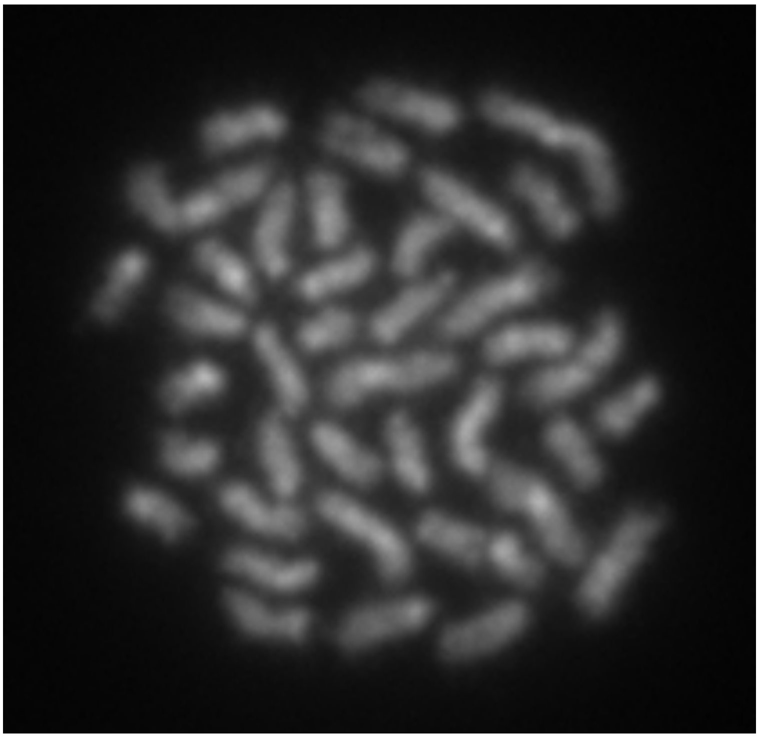

Supplementary Fig. S1. Metaphase spread from a *S. spinachia* male, showing the diploid chromosome number of 40.

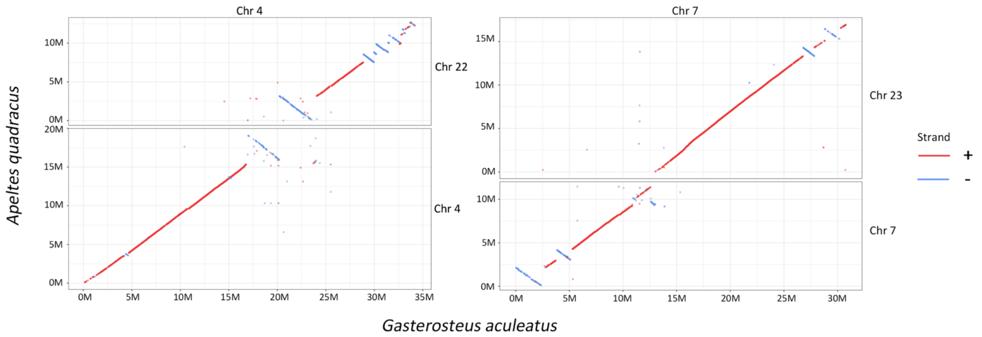

Supplementary Fig. S2. Synteny map of *G. aculeatus* chromosomes 4 and 7 compared with *A. quadracus*, based on coding region sequences using Mummer4 and nucmer.

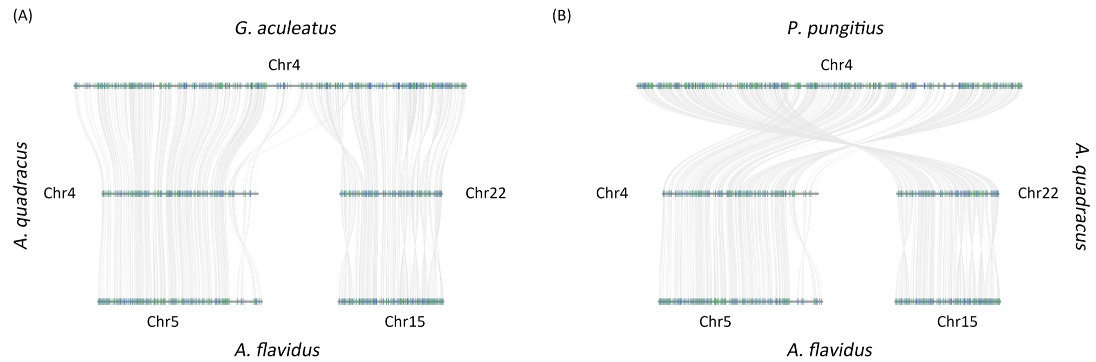

Supplementary Fig. S3. (A) Gene map of *G. aculeatus* chromosome 4 compared with *A. quadracus* and *A. flavidus*. (B) Gene map of *P. pungitius* chromosome 4 compared with *A. quadracus* and *A. flavidus*.

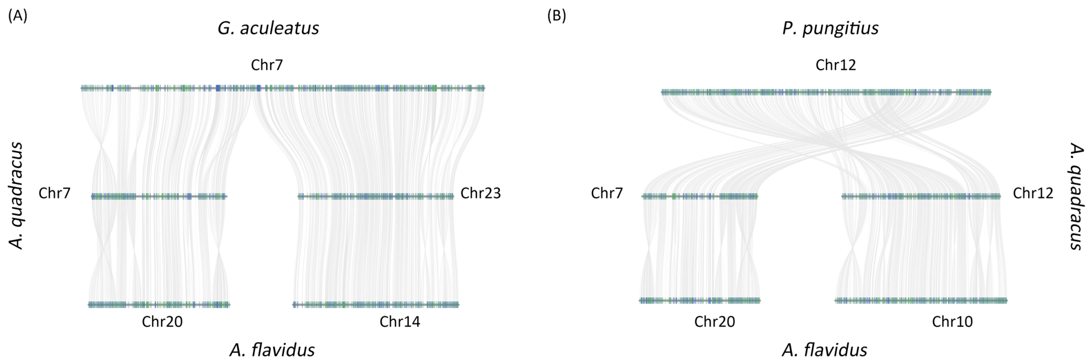

Supplementary Fig. S4. (A) Gene map of *G. aculeatus* chromosome 7 compared with *A. quadracus* and *A. flavidus*. (B) Gene map of *P. pungitius* chromosome 12 compared with *A. quadracus* and *A. flavidus*.

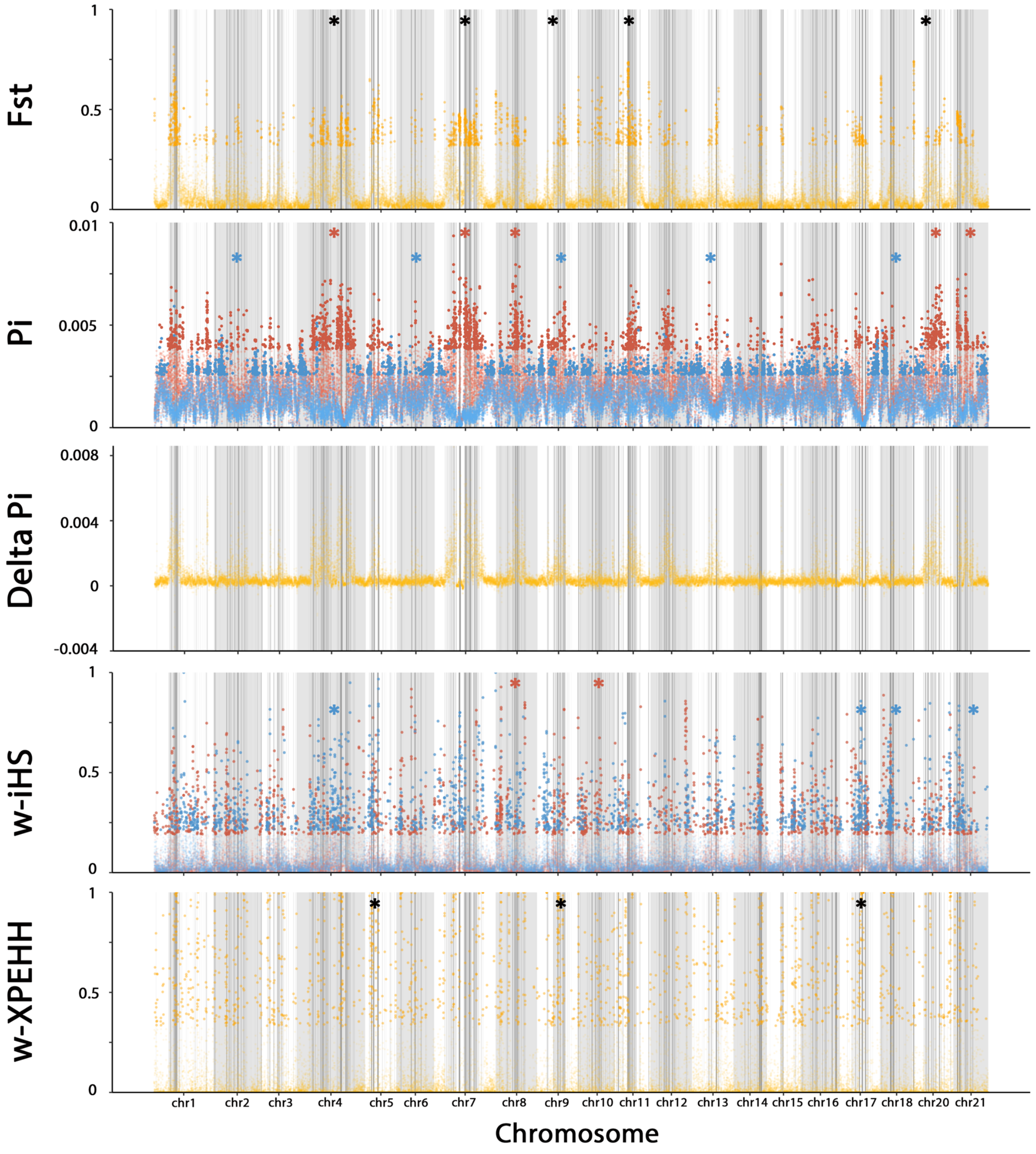

Supplementary Fig. S5. Signatures of selection in the Lake Washington freshwater and Puget Sound marine populations across the whole genome. All statistics were calculated in 20kb sliding windows with a step size of 10 kb. Dark grey bars indicate the genomic islands. From top to bottom: Fst distribution across the genome, with solid dots highlighting SNPs in the top 5% of genome-wide Fst; nucleotide diversity (Pi) of Lake Washington (red) and Puget Sound (blue) populations, with solid dots highlighting SNPs with the top 5% highest values of Pi in each population; differences of nucleotide diversity between the two populations. (Delta Pi = $Pi_{\text{Lake Washington}} - Pi_{\text{Puget Sound}}$); haplotype-based selection statistic iHS, with solid dots indicating the top 5% genome-wide outliers for Lake Washington (red) and Puget Sound (blue); and haplotype-based selection statistic XPEHH, with top 5% genome-wide outlier labeled in solid yellow dots. Asterisks represent chromosomes that show significantly greater evidence for selection in Lake Washington (red), Puget Sound (blue) or between the populations (black) than expected, given both the length of the chromosome and the number of genes on the chromosome, based on the standardized residuals from a chi-squared test (Supplementary Table S4).

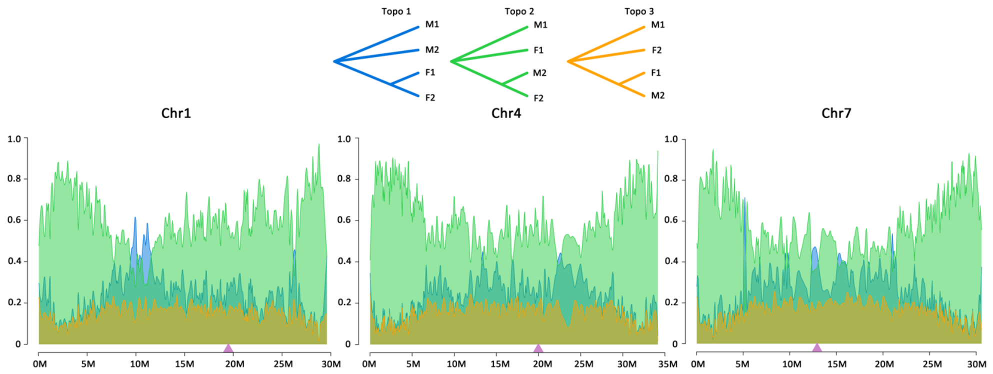

Supplementary Fig. S6. Topology weightings of marine and freshwater haplotypes in *G. aculeatus*. M represents marine haplotypes, while F represents freshwater haplotypes. M1 and F1 represent the major alleles in the respective populations, while M2 and F2 represent the minor alleles. Topo 1 represents the topology in which marine and freshwater ecotypes consistently diverge. Topo 2 and 3 represent topologies in marine and freshwater haplotypes that are not divergent. Purple triangles represent centromeres as well as the fusion points on chromosomes 4 and 7.

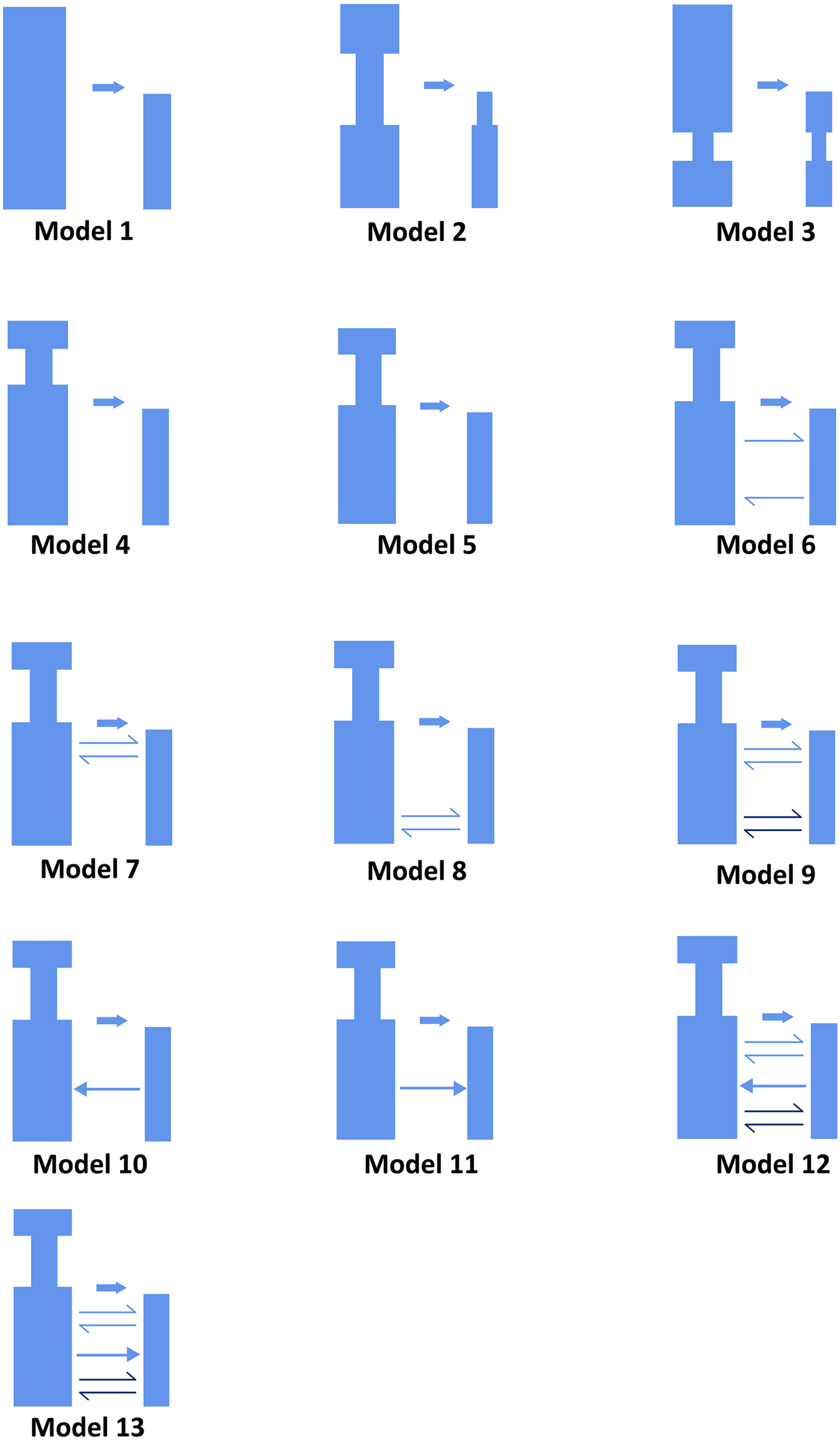

Supplementary Fig. S7. Models used in demographic modeling, with the Puget Sound population indicated on the left and the Lake Washington population indicated on the right: 1) constant population size; 2) two bottlenecks while splitting; 3) two bottlenecks after

splitting; 4) one bottleneck before splitting; 5) one bottleneck and splitting; 6) one bottleneck and splitting followed by a constant and reciprocal migration; 7) one bottleneck and splitting followed by an early reciprocal migration; 8) one bottleneck and splitting followed by a recent reciprocal migration; 9) one bottleneck and splitting followed by two reciprocal migration regimes; 10) one bottleneck and splitting followed by introgression from Lake Washington to Puget Sound; 11) one bottleneck and splitting followed by introgression from Puget Sound to Lake Washington; 12) one bottleneck and splitting followed by introgression from Lake Washington to Puget Sound and two reciprocal migration regimes; 13) one bottleneck and splitting followed by introgression from Puget Sound to Lake Washington and two reciprocal migration regimes.

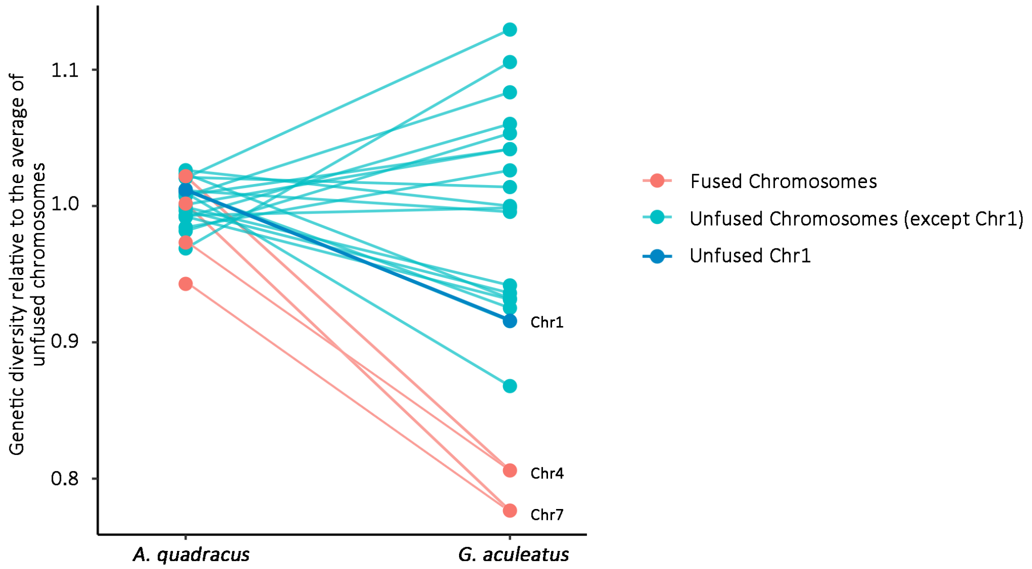

Supplementary Fig. S8 Comparison of genetic diversity between fused and unfused chromosomes in *A. quadracus* and *G. aculeatus*. Genetic diversity is a proxy for measuring the recombination rate on each chromosome and was calculated based on four-fold degenerate sites and normalized relative to the average diversity of unfused chromosomes. The two fused chromosomes (red) have lower genetic diversity relative to the average of unfused chromsomes (dark and light blue) in *G. aculeatus* as well as relative to their unfused homologues in *A. quadracus*, suggesting lower recombination rates on the fused chromosomes.

Supplementary Table S1. Sample information and accession numbers for sequencing data in this study.

Supplementary Table S2. QTL database used in this study and results for the chi-square test of QTL distribution on *G. aculeatus* chromosomes. All QTL are related to traits that differ between marine and freshwater ecotypes, with redundancies removed. Each QTL was examined to determine whether the phenotypic effect of the QTL was in the expected direction, based on the direction of divergence between the parental populations. The expected number of QTL with effects in the expected direction on each chromosome was calculated both by length in base pairs and number of genes on the chromosome and compared to the observed distributions in R using a goodness-of-fit test (chisq.test). Following Peichel and Marques (2017), chromosomes with significantly more QTL in the expected direction were identified by standardized residuals with a value larger than 3 in both comparisons and are highlighted in bold.

Supplementary Table S3. Distribution of gene transposition and gene duplication events on *G. aculeatus* chromosomes. The expected distribution of duplicated genes on each chromosome was calculated both by chromosome length in base pairs and the number of genes on the chromosome and compared to the observed distribution in R using a goodness-of-fit test (chisq.test). Chromosomes with significantly higher values than expected were identified by standardized residuals with a value larger than 3 in both comparisons and are highlighted in bold. There were too few gene transposition events to perform a similar analysis. Chromosome 19 (the sex chromosome) is omitted from these analyses.

Supplementary Table S4. Results of the chi-square test of distribution of signatures of selection on *G. aculeatus* chromosomes, including SNP numbers in genomic islands, nucleotide diversity (Pi), the proportion of iHS, and proportion of XPEHH. The expected distribution on each chromosome was calculated both by chromosome length in base pairs and number of genes on the chromosome and compared to the observed distribution in R through a goodness-of-fit test (chisq.test). Chromosomes with significantly higher values than expected were identified by standardized residuals with a value larger than 3 in both comparisons and are highlighted in bold. Chromosome 19 (the sex chromosome) is omitted from these analyses.

Supplementary Table S5. Results of the comparisons among the 13 demographic models, and the medians and 95% confidence interval of the parameters in the best-fitting model (model 9: one bottleneck and splitting followed by two reciprocal migration regimes).

Reference

Peichel CL, Marques DA. 2017. The genetic and molecular architecture of phenotypic diversity in sticklebacks. *Philos. Trans. R. Soc. Lond. B. Biol. Sci.* 372.
